# Case Report: Clinical and magnetic resonance imaging findings in intracranial hemangiosarcoma in a cat

**DOI:** 10.3389/fvets.2026.1919431

**Published:** 2026-09-18

**Authors:** Irene Marsellés-González, Livia Benigni, Emilie Cloup, Ricardo Fernandes

**Affiliations:** 1 Neurology and Neurosurgery Service, The Ralph Veterinary Referral Centre, Marlow, Buckinghamshire, United Kingdom; 2 Diagnostic Imaging Service, The Ralph Veterinary Referral Centre, Marlow, Buckinghamshire, United Kingdom; 3 Antech Diagnostics, Dick White Veterinary Specialists, Cambridgeshire, United Kingdom

**Keywords:** case report, cat, central nervous system, hemangiosarcoma, hemorrhage, intracranial

## Abstract

Hemangiosarcoma (HSA) is a highly malignant tumor of endothelial origin that is infrequently reported in cats, accounting for less than 2% of nonhematopoietic neoplasms. Feline HSA is typically classified into dermal and visceral subtypes, both of which exhibit metastatic potential. While HSA is the tumor most commonly reported to metastasize to the brain in dogs, intracranial involvement in cats remains rare, with only one previously documented metastatic case. This case report details the clinical presentation, magnetic resonance imaging (MRI) features, histopathological findings, and gross post-mortem brain changes in a cat diagnosed with intracranial hemangiosarcoma.

## 1 Introduction

The most commonly diagnosed central nervous system (CNS) neoplasms of cats are meningioma, glioma (1), and lymphoma (2). Pituitary tumors, although frequently diagnosed intracranial masses, are considered extra-axial and of non-CNS origin (3). Other tumor types affecting the CNS are considered rarer. Hemangiosarcoma (HSA) is a mesenchymal malignant neoplasm that affects dogs and cats, and has historically been thought to originate from vascular endothelial cells; more recent evidence suggests a possible derivation from pluripotent bone marrow progenitor cells (4). Dogs are affected far more commonly than cats, and the disease is rare in humans (4, 5). In cats, HSA accounts for less than 2% of nonhematopoietic neoplasms (6). Overall, HSA carries a poor prognosis in both dogs and cats (4).

Feline HSA is traditionally classified as dermal (cutaneous-subcutaneous) or visceral, with both forms occurring with similar frequencies. Tumors originating within the abdominal cavity have historically been associated with higher metastatic rates; however, recent reports suggest that cutaneous-subcutaneous forms may exhibit more aggressive biological behavior and greater metastatic potential than previously recognized (6); with both forms also appearing to have a poor prognosis (7).

In dogs, HSA is the tumor most commonly reported to metastasize to the brain (4). In contrast, intracranial involvement in cats is rare; however, both metastatic and primary intracranial hemangiosarcoma have been described (3, 4). A case report from 1994 described a cat with an inguinal subcutaneous HSA that subsequently developed cerebral metastatic lesions (8). Primary HSA of the CNS is uncommon in both humans and dogs, with approximately 30 cases reported in people and only a limited number documented in dogs. In cats, reports of primary intracranial HSA are exceedingly rare (9). To the authors’ knowledge, there are no documented cases of intracranial HSA in cats with detailed neuroimaging and histopathological correlation previously published.

This report aims to describe the clinical presentation, magnetic resonance imaging (MRI) findings, histopathological features, and gross brain pathology of a cat diagnosed with intracranial hemangiosarcoma.

## 2 Case description

A 6.5-year-old male neutered domestic short-haired (DSH) cat was referred to the Neurology Department at The Ralph Veterinary Referral Centre for evaluation of progressive right vestibular and diffuse forebrain signs. Clinical signs commenced one week prior to presentation. The owners noticed generalized ataxia, initially more pronounced in the pelvic limbs before progressing to the thoracic limbs. Over the following days, the patients’ condition deteriorated, manifesting dullness, rotatory nystagmus, and right-sided circling 24 h before referral. The cat had been previously healthy, was imported from Greece as a kitten, had supervised outdoor access with no known exposure to toxins, and was up to date with vaccinations and endo/ectoparasite preventatives.

General physical examination was unremarkable. The cat had a dark coat with no obvious integumentary lesions detected. Neurological examination revealed moderate to marked obtundation, a decerebellate posture with increased extensor tone in the thoracic limbs (more pronounced on the right), right-sided head tilt, severe non-ambulatory tetraparesis, and absent postural reactions in all four limbs. Cranial nerve assessment showed an absent menace response in the right eye, rotatory nystagmus (direction of the fast phase not recorded in the medical record), anisocoria with right miosis, and mild right ptosis, consistent with right-sided Horner’s syndrome.

The presence of a decerebellate posture and right-sided Horner’s syndrome indicated involvement of the brainstem and/or cerebellum beyond a pure central vestibular lesion. The right-sided head tilt, rotatory nystagmus, and right-sided circling, are consistent with a vestibular lesion (brainstem and/or cerebellar involvement). The absent menace response in the right eye was interpreted as most likely an afferent (visual pathway) deficit associated with the left forebrain, or ipsilateral cerebellum or brainstem. An ipsilateral facial (efferent) contribution could not be entirely excluded, although appeared less likely given that no other facial nerve dysfunction was perceived upon examination. Obtundation and absent postural reactions in all four limbs, and the overall clinical picture could support concurrent forebrain involvement; although this could as well be explained by a brainstem lesion. Neurolocalization was therefore consistent with multifocal disease involving the forebrain (lateralized to the left) and the right brainstem and cerebellum (central vestibular system).

A complete blood count revealed anemia (hematocrit 20%; reference interval [RI] 32–48%), eosinopenia (0 × 10^9^/L; RI 0.17–1.57 × 10^9^/L), and thrombocytopenia (137 × 10^9^/L; RI 151–600 × 10^9^/L). Packed cell volume was decreased (25%; RI 30–45%). Serum biochemistry was unremarkable, and coagulation profiles (activated partial thromboplastin time and prothrombin time) were within normal limits.

The cat was anaesthetized to undergo brain MRI (Figure 1). MRI demonstrated multiple, variably sized, intra-axial lesions predominantly located within the right cerebral hemisphere, with additional involvement of the left thalamus, brainstem, and a central and mildly right-sided cerebellar lesion. A difference in signal was noticed between the larger lesions and the small lesions. The smaller lesions were hyper- to isointense on T2-weighted (T2W) and fluid-attenuated inversion recovery (FLAIR) images, and isointense on T1W-weighted (T1W) images. Many showed mild to moderately contrast-enhancement, some were not enhancing (Figure 1). The larger lesions (>7 mm) were heterogeneous, of mixed signal intensity, and predominantly hyperintense to the corresponding normal parenchyma in T2W and FLAIR, slightly hypo, slightly hyper or isointense in T1 and showed complete, irregular rim of contrast enhancement (Figure 2). Most lesions showed complete signal void on T2*-weighted gradient-echo images (GRE), consistent with intralesional hemorrhage. Some showed an isointense central area surrounded by signal void. GRE was the sequence that enabled identification of the highest number of lesions, including very small lesions that were not identified in any other sequence. There was moderate to marked, perilesional, diffuse ill-marginated hyperintensity in T2W and FLAIR images, extending to the corona radiata, consistent with vasogenic oedema, or perilesional inflammation, resulting in right-to-left midline shift, caudal transtentorial herniation, and transforaminal herniation. Focal perilesional oedema was also noticed associated with some of the smaller lesions. Additionally, two small (approximately 0.5 and 0.2 cm), rounded, T2W, FLAIR, T1 and GRE hyperintense lesions with contrast-enhancement were identified within the right masseter and right temporalis muscles; these were not sampled. Thoracic radiographs were normal. Abdominal ultrasound revealed bilateral renal cysts. Infectious disease testing (IDEXX® snap test for Feline Immunodeficiency Virus [FIV] and Feline Leukemia Virus [FeLV] and for *Dirofilaria immitis*, *Borrelia burgdorferi*, *Ehrlichia* spp., and *Anaplasma* spp.; and Cryptococcal antigen serology) was negative. Toxoplasma IgG serology was positive (1:400), suggestive of prior exposure. Urinalysis was normal, and Baermann testing on feces yielded no larvae.

**Figure 1:**
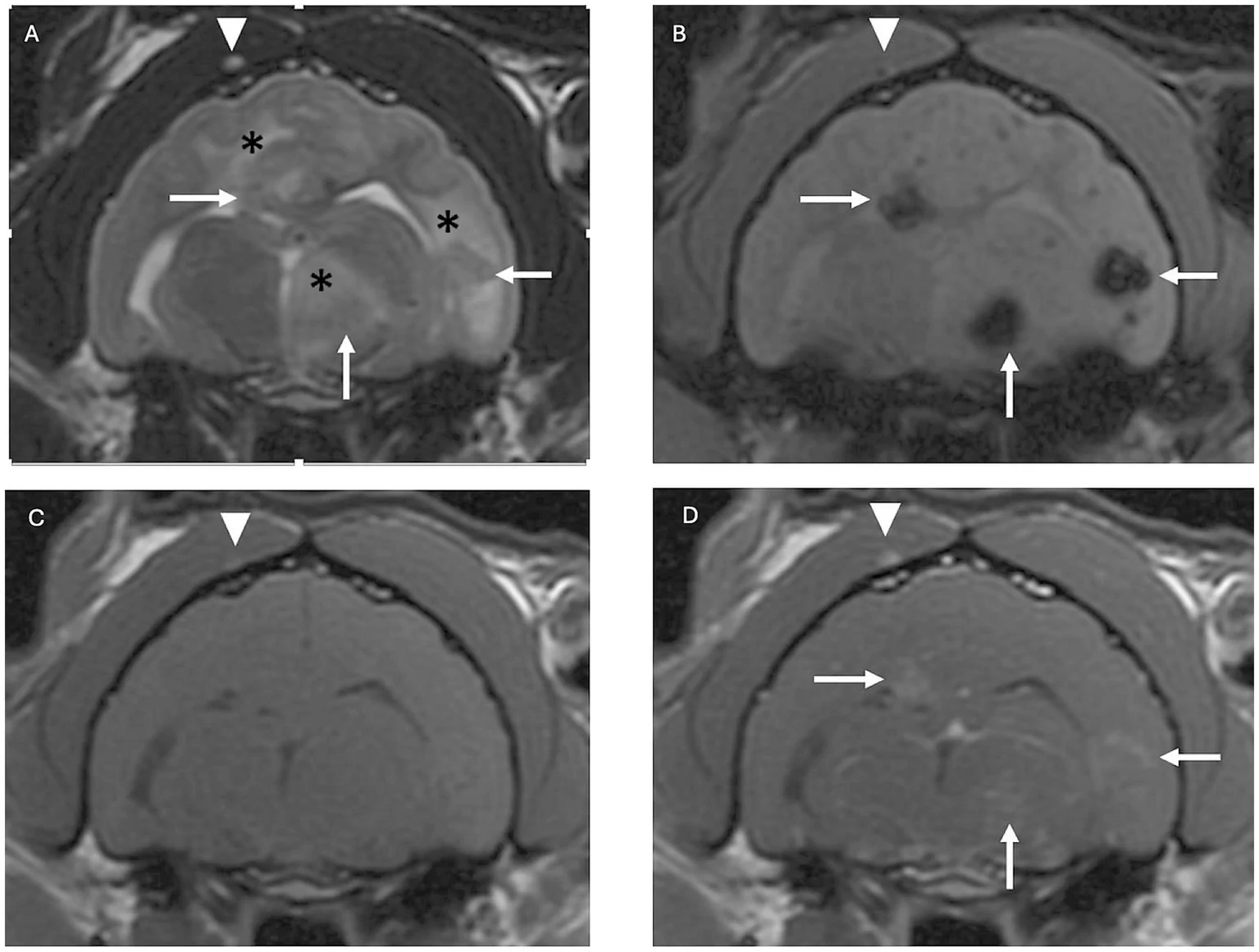
Transverse MRI images at the level of the thalamus. T2-weighted image shows ventricular asymmetry, a moderate to marked hyperintensity of the corona radiata, right cingulum and left thalamus (black asterisks), and isointense parenchymal lesions (white arrows) in the left thalamus, right corpus callosum and left temporal lobe **(A)**. Gradient echo image shows three larger (white arrows) and a dozen punctiform signal void **(B)**. No lesions are identified on the T1W-weighted image; the lesions identified in T2W and GRE are isointense on T1W **(C)**. T1-weighted post-contrast image shows irregularly marginated mild to moderate contrast enhancement corresponding to the location of the larger lesions found in GRE (white arrows); the punctiform lesions identified in GRE are not visible in T1W post-contrast. A small lesion in the right temporalis (white arrow head) muscle is hyperintense in the t2-weighted and t1-weighted images **(A,C)** and shows mild contrast enhancement **(D)**.

**Figure 2:**
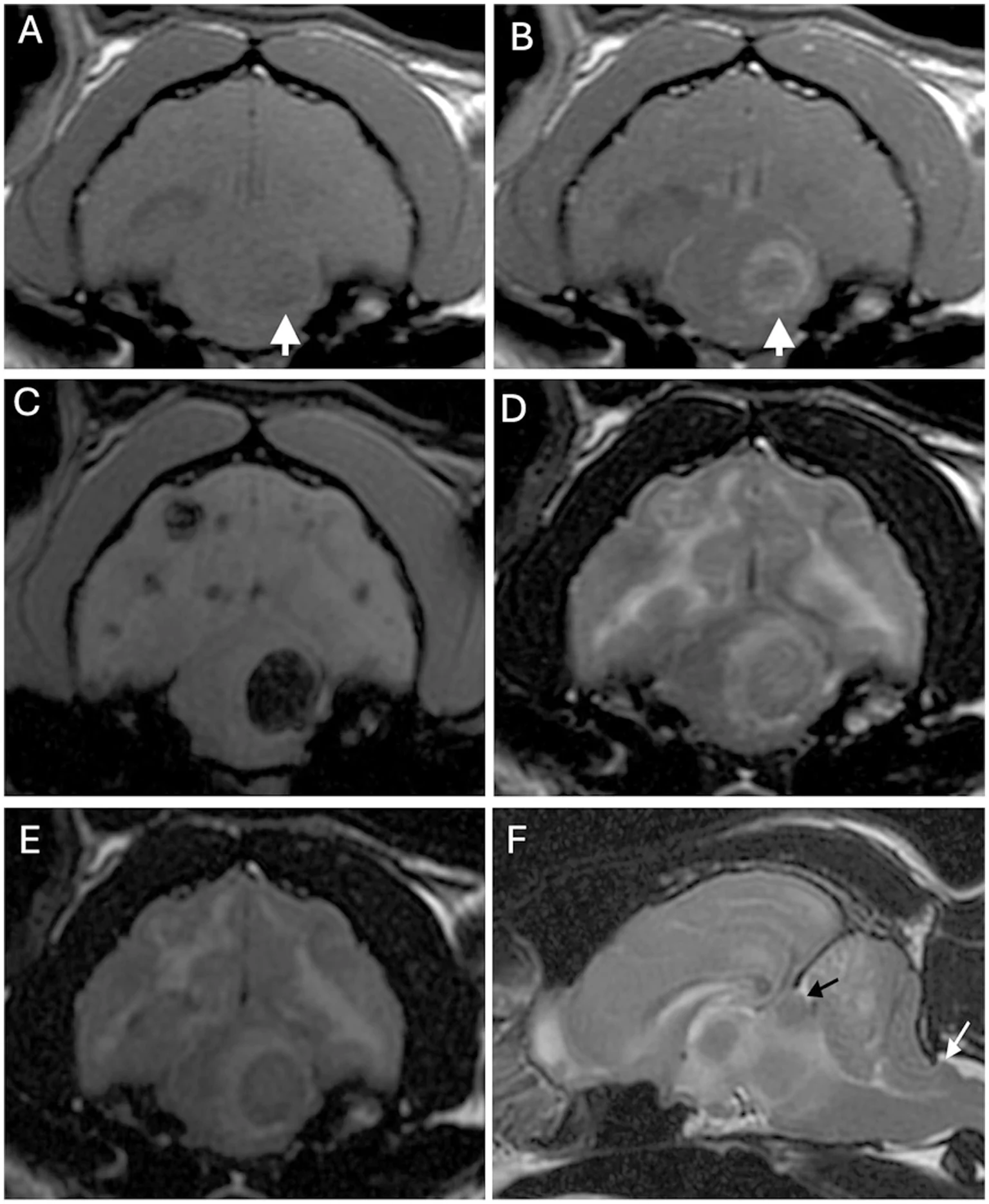
Transverse T1-weighted **(A)** and T1 post-contrast **(B)** images of the brain at the level of the occipital lobes show a large, slightly hypointense lesion with a complete rim of contrast enhancement located within the left pons. Transverse T2*-weighted gradient echo **(C)** enables the identification of very small lesions that are not identified in any other sequence throughout both occipital lobes. Transverse T2-weighted **(D)** and FLAIR **(E)** images of the brain show moderate-to-marked perilesional, diffuse, ill-defined hyperintensity, consistent with vasogenic oedema or perilesional inflammation affecting predominantly the internal capsule and corona radiata (more left-sided). **(F)** Sagittal T2-weighted image of the brain. The brain lesions are causing caudal transtentorial herniation (black arrow) and transforaminal herniation (white arrow).

Based on the clinical presentation and diagnostic findings, differential diagnoses included neoplastic disease (HSA, carcinoma), or parasitic hemorrhagic meningoencephalitis (e.g., aberrant migration of *Aelurostrongylus abstrusus*).

Upon recovery from anesthesia, the cat experienced a generalized tonic–clonic seizure and became progressively stuporous. Supportive therapy was initiated, including intravenous fluids (*Hartmann’s* solution at 2 mL/kg/h), oxygen-therapy (1–2 L/min), anti-epileptic medication (levetiracetam 20 mg/kg IV TID, midazolam constant rate infusion [CRI] 0.2 mg/kg/h, and dexmedetomidine CRI at 0.1–0.2 mcg/kg/h), broad-spectrum antibiotic (clindamycin 12.3 mg/kg IV BID), tranexamic acid 10 mg/kg IV TID, dexamethasone 0.2 mg/kg IV SID, mannitol 10% (0.5 g/kg TID for suspected increased intracranial pressure), and Advocate® spot-on solution (imidacloprid and moxidectin) while pending results infectious serologies and coprology.

Despite treatment, the cat experienced cardiorespiratory arrest 24 h after diagnostic investigations.

The owner consented to post-mortem brain examination, which was performed immediately after death. Grossly, the brain showed multiple dark red, friable, areas throughout the right olfactory lobe and adjacent right frontal lobe, and additionally at the level of the medullary pyramids and within the right ventral cerebellar hemisphere (Figure 3). Microscopically, the lesions affected both white and grey matters and represented an infiltrative, poorly demarcated mesenchymal neoplasm (Figure 3). The neoplastic cells were arranged in short streams and blood-filled tortuous vascular channels. The neoplastic cells were spindle shaped, intermediate to large sized, with poorly distinct cell borders and small amounts of pale eosinophilic cytoplasm. Nuclei were ovoid to irregularly shaped, with finely stippled chromatin and 1–4 large magenta nucleoli. The mitotic count was 6 mitoses per 2.37 mm^2^ (10 HPFs). Multifocal extensive hemorrhages replaced the neoplastic tissue. Overall, these histological features were consistent with hemangiosarcoma.

**Figure 3:**
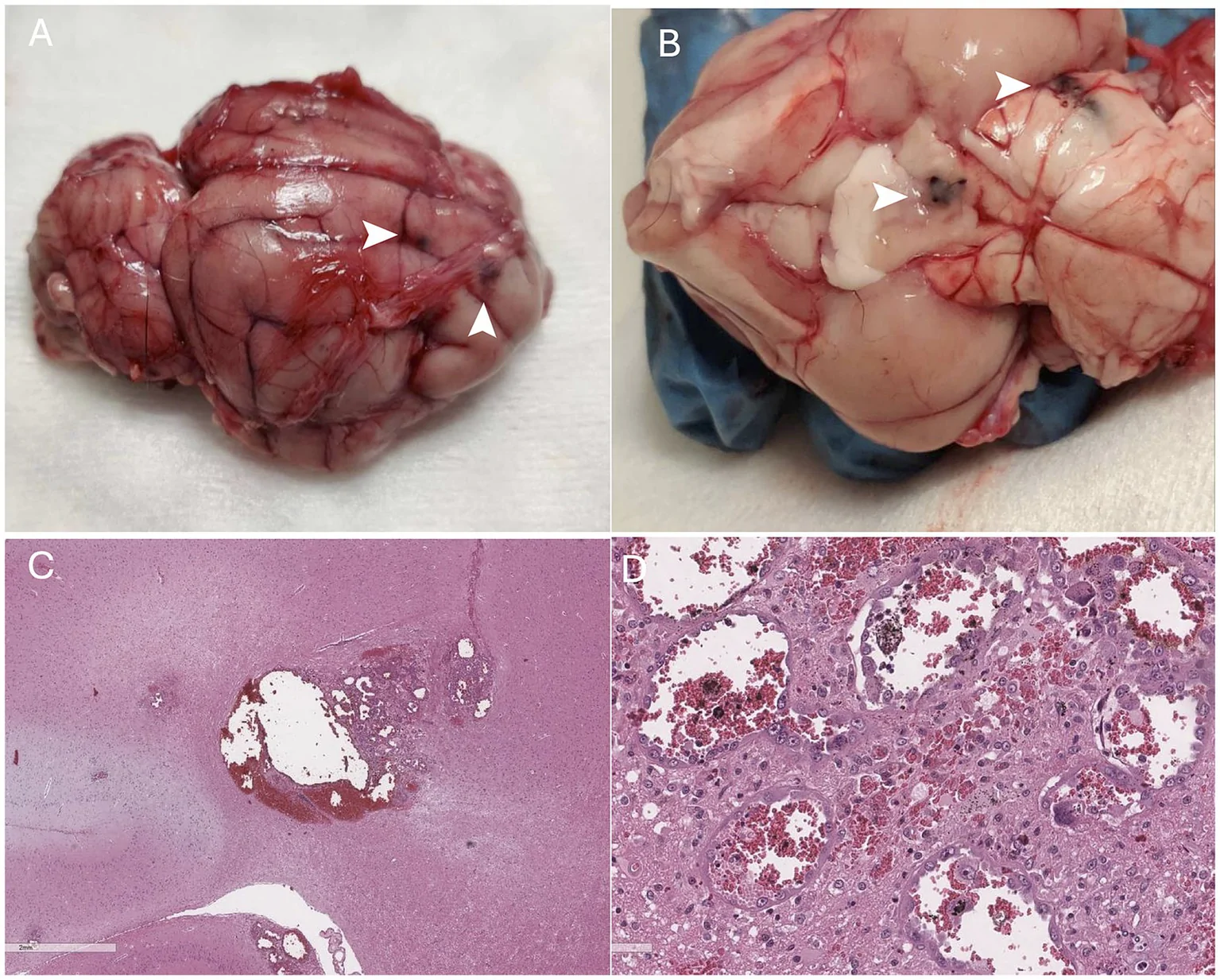
Cerebral hemangiosarcoma in a cat: Gross and histological features. Dorsolateral **(A)** and ventral **(B)** gross images of the brain showing two dark red, hemorrhagic, poorly demarcated cerebral lesions (arrow heads) within the right olfactory and frontal lobes **(A)** and at the level of the pyramids and the left cerebellar hemisphere **(B)**. **(C)** Low-power photomicrograph demonstrating an infiltrative neoplasm forming irregular, blood-filled vascular channels within the cerebral parenchyma (Hematoxylin and Eosin stain, ×10, whole slide digital scanning). **(D)** High-power photomicrograph showing atypical spindle to polygonal endothelial cells lining variably sized, anastomosing vascular spaces, with moderate nuclear pleomorphism (Hematoxylin and Eosin stain, ×40, whole slide digital scanning).

A time line of the clinical course is presented in Table 1.

**Table 1:** Timeline of clinical course and interventions.

Time point	Clinical event/finding	Intervention/result
Day 0	Onset of generalized ataxia (pelvic limbs > thoracic limbs)	Supportive care at home
Day 6	Dullness, rotatory nystagmus, right-sided circling	Assessment at referring practice followed by referral to neurology service
Day 7	Neurological examination and MRI performed	Multifocal hemorrhagic lesions; supportive therapy initiated
Day 7 (post-MRI)	Generalized tonic–clonic seizure upon recovery; progressive stupor	Antiepileptic drugs, mannitol, tranexamic acid, dexamethasone, fluids, oxygen
Day 8	Cardiorespiratory arrest	Death; immediate post-mortem brain examination performed

Timeline summarizing the key clinical events, diagnostic procedures, treatment, and outcome in a cat with intracranial hemangiosarcoma.

## 3 Discussion

Imaging characteristics of intracranial HSA have not been previously reported in cats. The MRI features in this case align with those described for canine CNS HSA, including multiple (rarely single) lesions with mixed intensities on T2W, FLAIR, and T1W sequences, susceptibility artifacts on T2*W images, mass effects, marked perilesional oedema, and frequent peripheral contrast enhancement (9).

A limited number of primary intracranial HSA cases have been documented in dogs. Recent reports, describing two dogs with solitary intracranial HSA (presumed primary, with no extracranial disease identified on staging) and treated surgically, indicate that imaging features did not differ from those of metastatic disease (10). Likewise, in this case, MRI alone could not differentiate primary intracranial tumor from metastatic disease. While no overt extracranial metastases were identified on thoracic radiographs or abdominal ultrasound, two small T2W hyperintense, contrast-enhancing lesion were noted in the right masseter and right temporalis muscles. These lesions were not biopsied, and a full-body post-mortem examination was not performed, precluding exclusion of an extracranial primary tumor with intracranial metastasis. Furthermore, the cat’s dark-pigmented coat may have obscured a cutaneous primary HSA, constituting an additional limitation.

The bilateral renal cysts were interpreted as most likely representing, bilateral polycystic kidneys. Polycystic kidney disease (PKD) is an inherited condition affecting approximately 6% of cats (11). Associations between PKD and vascular tumors have been noted in other species, such as a donkey with concurrent metastatic HSA and PKD (12). In humans, links between PKD and vascular malformations or hemangiomas have been reported, with vascular endothelial growth factor hypothesized as a potential pathophysiological mechanism (13, 14). Certainly, a post-mortem examination of the kidneys would have been necessary to reach a definitive diagnosis and rule out cystic neoplasia.

Primary CNS angiosarcoma, the human counterpart of HSA, is similarly rare, with approximately 25–40 cases documented in the literature. It most commonly affects adults and frequently presents with acute neurologic signs secondary to intratumoral hemorrhage. MRI characteristics closely mirror those observed in this feline case and in canine CNS HSA, including heterogeneous T1W and T2W signal intensity due to blood products of varying ages, prominent susceptibility artifact on gradient-echo or susceptibility-weighted sequences, moderate-to-marked perilesional vasogenic edema with mass effect, and irregular peripheral or heterogeneous contrast enhancement (15, 16). Prognosis remains poor despite multimodal therapy, with rapid clinical decline often observed, highlighting the aggressive nature across species. Inclusion of HSA in the differential diagnosis for hemorrhagic intracranial lesions in cats is therefore warranted, paralleling considerations in human neuro-oncology. Moreover, other differential diagnosis for multiple hemorrhagic foci in the feline brain include coagulopathies, hypertensive encephalopathy, and infectious/parasitic diseases. Among parasitic etiologies, aberrant migration of nematodes such as *Gurltia paralysans*, *Angiostrgongylus* spp., and *Aelurostrongylus abstrusus* should be considered (17). Tinoco et al. described the first reported case of fatal hemorrhagic meningocencephalomyelitis in a kitten due to ectopic localisation of adult *Aelurostrongylus abstrusus* within the central nervous system, causing multifocal hemorrhagic necrosis and subarachnoid hemorrhages (17). Although uncommon, these parasitic conditions reinforce the need to include infectious etiologies in the differential diagnosis of multifocal hemorrhagic intracranial lesions in cats, particularly in young or immunocompromised individuals.

Tranexamic acid (TXA) is a synthetic lysine-analog antifibrinolytic agent, increasingly used in veterinary emergency and critical care for the management of hemorrhage associated with trauma, neoplasia (including hemangiosarcoma), and hyperfibrinolysis. Typical reported doses in dogs and cats are 10–20 mg/kg intravenously. Retrospective data support its use in critically ill dogs and cats with neoplasia or bleeding disorders, although evidence specific to intracranial hemorrhage in cats remains limited (18). In the present case TXA was administered empirically because of suspected ongoing intracranial hemorrhage; the rapid clinical deterioration precluded any meaningful assessment of its therapeutic effect.

To the authors’ knowledge, this is the first report of feline intracranial HSA with detailed MRI description and histopathological correlation, a condition that is exceedingly rare in cats. Interestingly, a number of small lesions were only visible in GRE, making this sequence an essential sequence to include in the brain MRI standard protocol. The MRI appearance mirrors that in canine CNS HSA and does not allow differentiation between primary and metastatic disease. A further limitation is that only the brain was examined post-mortem; the cervical spinal cord and neck were not imaged or sampled. The first-order (central) sympathetic pathway descends through the brainstem into the cervical and cranial thoracic spinal cord (T1–T3), after which the second-order (preganglionic) neurons exit the spinal cord and travel through the thorax and cervical region. Consequently, an undetected lesion affecting the right side of the cervical spinal cord or the ipsilateral preganglionic sympathetic pathway in the neck cannot be entirely excluded as a possible contributor to the right-sided Horner’s syndrome. While the intracranial findings (mass-effect-related distortion of the thalamus and brainstem) provide a plausible central explanation, the absence of cervical imaging and necropsy means a concurrent or alternative cervical lesion remains a theoretical possibility. Although no definitive extracranial primary was confirmed, metastatic etiology cannot be ruled out due to unsampled masseter and temporalis lesions, incomplete post-mortem evaluation, and potential for undetected cutaneous disease. It is also possible that some of the smaller lesions visible only on GRE represented pure hemorrhagic foci secondary to tumor-related microangiopathy or coagulopathy rather than discrete neoplastic deposits, a phenomenon documented in canine metastatic HSA (19). This case adds to the sparse literature on feline CNS hemangiosarcoma and underscores the need to include this neoplasm in the differential diagnosis for cats with acute or progressive hemorrhagic intracranial lesions.

### 3.1 Owner’s perspective

The owners elected post-mortem examination of the brain to better understand the underlying cause and consented to publication of the findings to raise awareness of this condition and potentially help other animals in the future.

## Data Availability

The datasets presented in this article are not readily available because this is a case report based on clinical data. Requests to access the datasets should be directed to the corresponding author.
